# Mucosal Melanoma in the Head and Neck Region: Different Clinical Features and Same Outcome to Cutaneous Melanoma

**DOI:** 10.1155/2013/586915

**Published:** 2013-05-16

**Authors:** Faruk Tas, Serkan Keskin

**Affiliations:** Institute of Oncology, University of Istanbul, Capa, 34390 Istanbul, Turkey

## Abstract

Mucosal melanoma (MM) in the head and neck (H&N) is relatively rare and behaves in distinct pattern from cutaneous melanoma (CM). We performed this study to define clinical characteristics and outcomes of patients and emphasize MM differences from CM. Forty-one patients with MM located in H&N were assessed. 94 CM patients originated from H&N region were also used for comparison. Patients had oral cavity (51%) and sinonasal location (49%).The median age was 60 years and gender distribution was equal. Thirty-two (78%) patients had localized stage, four (10%) patients had regional lymph node metastasis, and five (12%) patients had distant metastasis. The 1- and 5-year overall survival rates were 81% and 58%, respectively. Outcomes were similar between sinonasal and oral cavity patients (*P* = 0.67). Advanced disease was the significant prognostic factor for outcome (*P* = 0.03). MM patients are older (*P* = 0.008) and more diagnosed as a localized disease patients at presentation than those with CM (*P* = 0.06). Overall survival rates were identical in patients with MM and CM (*P* = 0.53). In conclusion, despite different clinical features, outcome was identical in patients with MM and CM located in the H&N region.

## 1. Introduction

Melanomas develop from melanocytes which are derived from the neural crest and are widely distributed throughout all cutaneous, ocular, and most of the mucosal surfaces. Cutaneous melanomas (CMs) are much more prevalent than noncutaneous melanomas, which are comprised of mucosal, ocular, and unknown primaries [1]. The annual age-adjusted incidence of noncutaneous melanomas was reported 0.7 per 100 000 persons. The vast majority of these tumors (approximately 80%) were ocular in origin. The remaining arose in the mucosa, resulting in an annual incidence of only 0.15 per 100 000 persons and accounting for nearly 3-4% of all melanoma diagnosed yearly [1, 2]. Likewise, Cancer Control Agency of British Columbia revealed a similar 4.5% of all melanoma patients who were diagnosed with a mucosal primary tumor [1, 3]. Based on the analyses of the National Cancer Data Base (NCDB) that comprised 84 836 cutaneous and noncutaneous melanoma cases, the percentages of mucosal melanoma (MM) were 1.3% [1, 4]. 

While it is a rare neoplasm of melanocytic origin, the head and neck (H&N) are the most common sites for MM, representing nearly half of MMs from all sites [1, 5, 6]. Additionally, it also accounts for approximately 1-2% of all melanomas and 2–8 of all H&N melanomas. It involves in decreasing order of frequency the sinonasal cavity (50%), oral cavity (45%), and other sites (5%) such as pharynx, larynx, and upper esophagus [1, 5, 6]. In other words, the most common site of occurrence for MM is the upper aerodigestive tract, most commonly the sinonasal area and oral cavity.

Up to the present time, approximately several hundred cases have been reported in a small number of retrospective series as well as case reports [5, 6]. Besides its rarity, H&N MM is also known as an aggressive neoplasm [1, 5, 6]. No prospective trials of patient management have yet been performed. Owing to the small size of most reported series and their retrospective nature, the effect of various treatment strategies on disease control and survival has been difficult to assess. The optimal management of H&N MM is not well defined. Treatment recommendations for H&N MM are based on small, retrospective studies often reporting very heterogenous patient groups collected over prolonged time periods. However, in light of these insufficient literature data, MM has a very poor prognosis and significant worse outcomes than CM, with 5-year disease-free survival rates ranging from 0 to 20%, with poor local as well as distant control.

In this paper, we review the clinical features of H&N MM in general and then provide a more detailed discussion concerning the presentation and management of tumors originating in this specific anatomic location compared with the CM originated from same localization.

## 2. Patients and Methods

The records of the adult cases of MM which have been pathologically proven at the Institute of Oncology, Istanbul University between January 2000 and December 2010 were retrieved from the cancer registry for review of the clinic-pathological features and outcome. We used the data regarding patients with CM obtained from another study of ours published in 2006 [7]. It contains the clinical records of 94 CM patients who were diagnosed and prospectively followed up at our Institution during a 12-year period (from January 1991 and December 2003) and then have been reviewed.

Staging of MM was proposed by Ballantyne [8]. Stage I tumors are confined to the primary site, stage II tumors have spread to regional lymph nodes, and stage III tumors represent distant metastasis. For CM, staging was determined according to the American Joint Committee on Cancer (AJCC) staging system. In situ melanoma patients were not included in the study. 

Surgery was the main treatment modality for localized and locoregional diseases (regional lymph node positive) of melanoma patients located in H&N region. Radiotherapy was used for patients not eligible to surgery for local therapy and regional lymph node metastasis. Adjuvant therapy with interferon (alpha2b) was given in patients with regional lymph node metastasis during 1 year. In patients with resected regional lymph node metastasis, radiotherapy was given as adjuvant therapy intent. The chemotherapy including dacarbazine, temozolomide, cisplatin, and fotemustine was used for patients with distant metastatic disease. Regimens of single or combined chemotherapy were selected based on performance status of patients and extension of disease. Nonresponder patients to chemotherapy had treated with second-line chemotherapy if they were good performance status. 

Overall survival was analyzed by the Kaplan-Meier method. Overall survival time was calculated from the onset of primary melanoma diagnosis to the date of the last followup at which patients were still alive or to the date of death. Univariate analysis of the association between the prognostic factors and survivals was performed using log-rank tests. Values of *P* < 0.05 were considered to be significant.

## 3. Results

Forty-one patients with MM and ninety-four patients with CM localized in the H&N were included in the study. The clinical characteristics are shown in Table 1. 

### 3.1. MM in the H&N Region

A total of 41 patients were diagnosed with MM and divided to two main groups that are distributed equally to oral cavity (*n* = 21, 51%) and sinonasal location (*n* = 20, 49%) consisting of nasal cavity (*n* = 11) and paranasal sinus (*n* = 9) (Table 1). The median age of all MM patients was 60 years and age was similar in patients with sinonasal and oral cavity locations (57 versus 63 years, resp.; *P* = 0.64). Likewise, gender distribution of the patients was equally (nearly 50%) and similar (*P* = 0.64). Thirty-two (78%) patients had localized stage (stage I), four (10%) patients had regional lymph node metastasis (stage II), and five (12%) patients had distant metastasis (stage III). Similar distributions were found in MM patients with sites of sinonasal and oral cavity (*P* = 0.56). 

**Figure 1:**
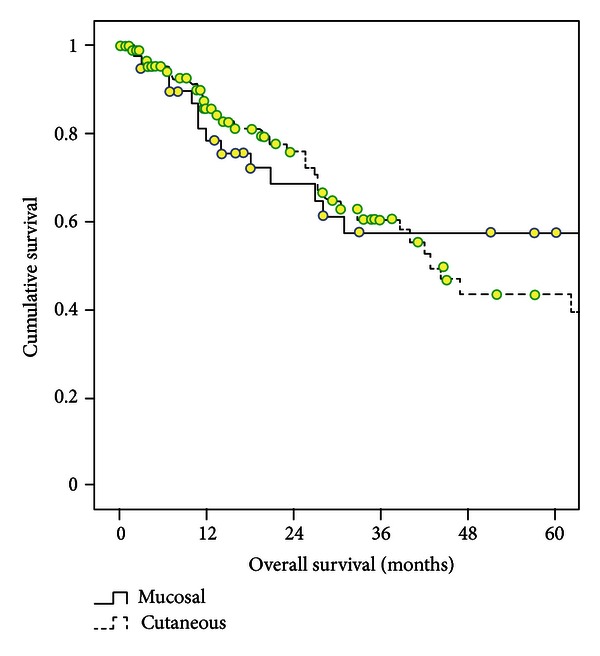
Survival curves in patients with MM and CM located in H&N region (*P* = 0.53).

**Figure 2:**
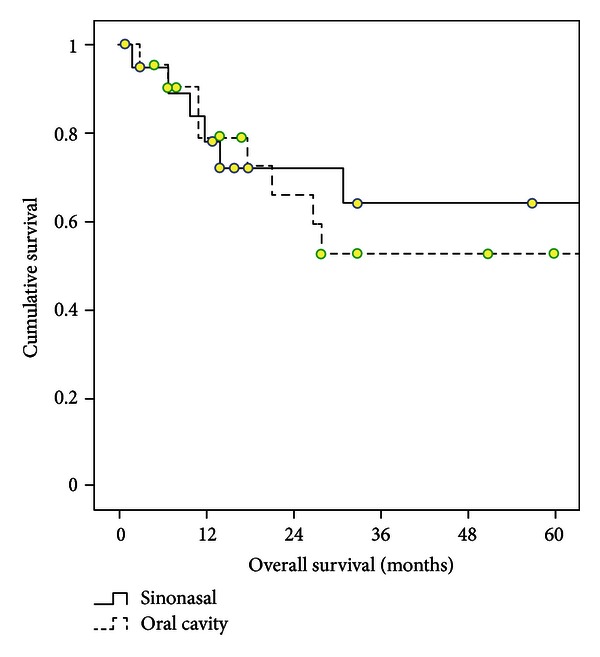
Survival curves in patients with MMs located in H&N region (*P* = 0.67).

An estimated 1- and 5-year overall survival rates in 41 MM patients were 81% and 58%, respectively. (Figure 1). The 1- and 5-year survival rates were 84% and 64% for patients with sinonasal and 79% and 53% for oral cavity, *P* = 0.67 (Figure 2). Advanced disease (stage II and III) at presentation was the significant prognostic factor for outcome in Cox regression analysis (*P* = 0.03) (Table 2). This finding was originated from only patients with oral cavity (*P* < 0.001), not sinonasal localization (*P* = 0.51). However, age and gender of MM patients did not affect survival. 

### 3.2. CM in the H&N Region

Ninety-four CM patients localized to H&N region are shown in Table 1. Face was the most common anatomic site (*n* = 50, 53%) which was followed by scalp (*n* = 26, 28%) and ear region (*n* = 18, 19%). The median age was 54 years and the patients localized to face was older (59 years, *P* = 0.001) than both scalp (46 years, *P* = 0.011) and ear region (47 years, *P* = 0.012) originated patients. Similarly, gender of patient was found to be statistically significantly different among CM patients (*P* = 0.005). Scalp localized MM patients were more male (81%) than face (42%, *P* = 0.001) and ear region (50%, *P* = 0.03) sited lesions. The distribution of disease stage was found to be significantly different in the CM patients (*P* < 0.001). While vast majority of CMs originated from face were localized disease (84%), advanced disease was found in most of the patients with ear region (72%) and equally (46%) in scalp region patients. Therefore, statistically significant differences were found to be face versus scalp (*P* = 0.002), face versus ear region (*P* < 0.001), but no scalp versus ear region (*P* = 0.47). 

**Figure 3:**
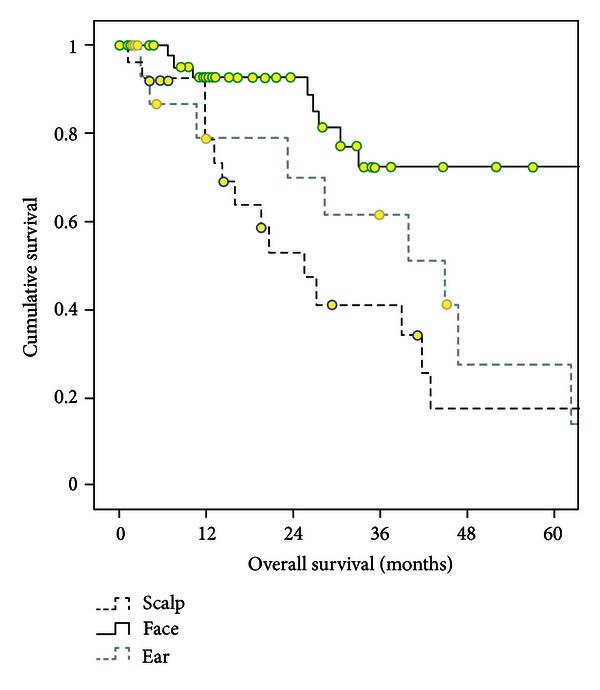
Survival curves of patients with CMs located in the head (face versus scalp, *P* < 0.001; face versus ear, *P* = 0.012; and ear versus scalp, *P* = 0.40).

The 1- and 5-year overall survival rates in 94 CM patients were 86% and 43%, respectively (Figure 1). The 1- and 5-year survival rates were 93% and 72% for patients with tumors localized in face, 79% and 27% for ear region, and 78% and 17% for scalp region (face versus scalp, *P* < 0.001; face versus ear, *P* = 0.012; and ear versus scalp, *P* = 0.40) (Figure 3). At presentation, advanced disease (stage III and IV, AJCC) was the significant prognostic factor for survival (*P* = 0.001) (Table 2). This difference was originated from only patients located on scalp region (*P* < 0.004), not facial (*P* = 0.9) and ear region (*P* = 0.64). Likewise, we found that being male patients is a poor prognostic factor in patients with CM in H&N region (*P* = 0.034) and in lesions located in ear region (*P* = 0.034). However, age of patient did not affect survival. 

### 3.3. Comparisons of Melanoma Types

Median ages of patients were found to be statistically significantly different between MM and CM patients (*P* = 0.008) (Table 1). Patients with MM were older than those with CM (median ages 60 versus 54 years). Contrarily, for gender distribution, any significant differences among melanoma subsets were not determined (*P* = 0.56). Additionally, a more percentage of MM was diagnosed as localized disease at presentation than that of CM (78% versus 65%, *P* = 0.06). 

The 1-, and 5-year survival rates in patients with MM and CM were 81% and 86% versus 58% and 43%, respectively (Figure 1). No significant difference was found between different anatomic locations (*P* = 0.53).

## 4. Discussion

Melanocytes in the mucosa of the upper aerodigestive tract are biologically identical to melanocytes in the skin as they are all neural crest derived [1, 5, 6]. Melanocytes are normally found throughout the mucosa of the aerodigestive tract compared with other sites, less frequently found in oral mucosa which may explain the lower incidence of melanomas in this area [5, 6]. In most reports of H&N MM, the nasal cavity (50%) and oral cavity (45%) were the most common sites [1, 5, 6]. The majority of sinonasal MM arise from the nasal cavity (80%) and the rest from the sinuses (20%) [5]. The maxillary sinus was the most commonly involved site among the paranasal sinuses (75%). We found similar results in accordance with the literature. Primary MMs of the pharynx and larynx are exceedingly rare (5%). 

In majority of series including this study, gender was equally balanced, although others have described a slight male and female predominance [5, 6]. In the literature, most of MM patients were diagnosed after the age of 40 years, typically one-two decades later than CM patients [5]. In this study, we found similar results; patients with MM are older than than others with CM by 6 years (median 60 versus 54 years, *P* = 0.008). Generally, oral cavity melanoma appears to occur at a younger age than sinonasal MM [5]. Such a difference was not found in our study. MM of the H&N in the very young is extremely uncommon [5]. The youngest patient was 35 years old in this study.

MM is occasionally staged according to the American Joint Committee on Cancer (AJCC) staging system [1, 5, 6]. The absence of a dermal epidermal junction in mucosa and the presence of ulceration in most of MM lesions lead to problem to use of this system; therefore, the current AJCC staging system has little relevance to the prognosis of MM. Nowadays, the most commonly used staging system for MM is that proposed by Ballantyne [8]. There is still the lack of a validated clinicopathologic staging system [1, 5]. 

Patients with oral cavity present a significantly higher incidence of cervical lymph node metastases compared with sinonasal MM patients [5]. The incidence of neck node involvement at presentation was 25% and 6–10% for patients with oral cavity and sinonasal MM, respectively [5]. Similar results were found in this paper (%14 for oral cavity, 6% for sinonasal, and 10% for total MM).

Similarly, at admission, distant metastases are relatively rare, less than 10% [5]. In general, no clear difference between oral cavity and sinonasal MM in the literature is found. In this study, this rate was 12% and, we also found similar findings among different sites of lesions. The common sites of distant metastasis are the lungs (33%) and brain (14%), with multiple sites being involved in 33% of cases in the literature [5]. In this study, we found that only five patients with distant metastasis had lung (3 patients), liver (2 patients), brain (1 patient), bone (1 patient), and multiple sites (2 patients). 

Generally, all MMs appear to be seen at a more advanced stage, behave more aggressively, and have a much worse overall prognosis and outcome [1, 5, 6]. Likewise, H&N region MM is also known as aggressive and recurrent neoplasm and therefore, it has poor prognosis. The 5-year overall survival rates vary between 17.1 and 35.1% [5, 6]. Tumor originated from the nasal cavity have the best outcomes with a reported 5-year survival of 15–30% compared with 12.3% for oral cavity MM and 0–5% for MM of the nasal sinuses [5, 6]. These ominous features are the result of an intrinsic biological aggressiveness in addition to that the diagnosis is usually made at a more advanced stage resulting from the relative inaccessibility of the occult anatomic locations of the lesions and infrequently early symptoms [5, 6]. However, we found that patients located in sinonasal region had survived similar to those with oral cavity because of presence of large population located in paranasal sinus.

The anatomic site of the primary CM correlated significantly with survival. Patients located in H&N region with CM are associated with a poorer prognosis among melanoma patients. Although H&N MM patients had poor prognosis, in this study, no significant difference was found between MM and CM patients (*P* = 0.53). Our patients with MM had significantly favorable outcome because of the fact that majority of the patients had localized disease. Therefore, this favorable outcome is balanced with survival available from CM patients. 

In the literature, effects of the prognostic factors on survival remain controversial [1, 5, 6]. Prognostic factors associated with unfavorable outcome include advanced stage, male sex, and older age in majority of the available literature [1, 5, 6]. However, others found that the patients' age, sex, site of primary tumor, and presence of regional nodal disease showed no influence on survival rates [1, 5, 6]. However, presence of distant metastasis was also found to affect overall prognosis. Patients with metastatic disease had much poorer survival [1, 5, 6]. In this study, while we found that age and gender of patients did not affect survival, advanced disease (stage II and III) at presentation was the significant prognostic factor for patient outcome. This finding was originated from only patients with oral cavity (*P* < 0.001), not with sinonasal localization. 

Because treatment recommendations for H&N region MM are based on small, retrospective studies often reporting very heterogenous patient groups collected over prolonged time periods and the lack of an agreed or accurate staging system, the optimal management of H&N MM is not well defined [1, 5, 6]. In these patients the effect of various treatment strategies on disease control and survival has been difficult to permit a comprehensive assessment [1, 5, 6]. In localized patients, surgery alone and in combination with radiotherapy has been advocated as definitive therapy. Despite its radioresistant nature of tumor, radiation therapy can be used if complete surgical excision may not be possible in many cases due to the extent of disease at presentation [5]. 

In conclusion, MM originated from H&N region did not share same clinicopathologic characteristics with CM. However, the survival rates seem identical. There are not widely accepted prognostic and predictive factors in patients with H&N region MM. Additionally, there is no effective systemic therapy for metastatic stage and novel therapies are urgently required. Hopefully, with a better understanding of the difference between melanomas subtypes and biologic factors influencing melanoma treatment, the survival of these patients will be improved.

## Figures and Tables

**Table 1:** Characteristics and distribution of various parameters depending on melanoma patients.

Site of melanoma	Age of patients median (range)	Gender of patients		Stage of disease		
		Male	Female	Locally	Lymph node positive	Distant metastatic
Mucosal melanoma						
Sinonasal	57 (35–79)	9 (45%)	11 (55%)	16 (80%)	1 (5%)	3 (15%)
Oral cavity	63 (38–84)	11 (52%)	10 (48%)	16 (76%)	3 (14%)	2 (10%)
*P* value	0.644	0.636			0.555	
Cutaneous melanoma						
Face	59 (17–104)	21 (42%)	29 (58%)	42 (84%)	8 (16%)	0 (0%)
Scalp	46 (18–72)	21 (81%)	5 (19%)	14 (54%)	6 (23%)	6 (23%)
Ear	47 (24–74)	9 (50%)	9 (50%)	5 (28%)	12 (67%)	1 (5%)
*P* value	0.001	0.005			<0.001	
Mucosal melanoma, total	60 (35–84)	20 (49%)	21 (51%)	32 (78%)	4 (10%)	5 (12%)
Cutaneous melanoma, total	54 (17–104)	51 (54%)	43 (46%)	61 (65%)	26 (28%)	7 (7%)
*P* value	0.008	0.558			0.062	

**Table 2:** Univariate analyses of various parameters depending on melanoma patients.

Site of melanoma	Age of patients 〈60 years〉	Gender of patients Male versus Female	Stage of disease Locally versus Advanced*
Mucosal melanoma			
Sinonasal	0.637	0.470	0.509
Oral cavity	0.253	0.858	<0.01
Total	**0.239**	**0.603**	**0.029**
Cutaneous melanoma			
Face	0.524	0.471	0.898
Scalp	0.304	0.993	0.004
Ear	0.320	0.034	0.643
Total	**0.784**	**0.034**	**0.001**

*Contains both regional lymph node metastasis and distant metastasis.
